# HCAR3 and Kynurenic Acid in Cancer: A Promising Axis of Immunometabolic Regulation or a Scientific Mirage?

**DOI:** 10.3390/ijms26136269

**Published:** 2025-06-28

**Authors:** Katarzyna Walczak, Dorota Krasowska

**Affiliations:** 1 Laboratory for Immunology of Skin Diseases, Chair and Department of Dermatology, Venereology and Paediatric Dermatology, Medical University of Lublin, Radziwillowska 11, 20-080 Lublin, Poland; 2 Chair and Department of Dermatology, Venereology and Paediatric Dermatology, Medical University of Lublin, Staszica 11, 20-081 Lublin, Poland

**Keywords:** HCAR3, cancer, kynurenine pathway, kynurenic acid

## Abstract

The hydroxycarboxylic acid receptor (HCAR) family belongs to G-protein-coupled receptors (GPCRs) implicated in a diverse array of physiological and pathological mechanisms. Kynurenic acid, a metabolite of the tryptophan catabolic pathway, has been proposed as a putative ligand of HCAR3. This receptor, among other HCARs, has garnered particular attention due to its exclusive expression in humans and closely related primates, and its emerging role in immunometabolic regulation. This review focuses on the potential role of HCAR3 in cancer initiation, progression, and metastasis. Moreover, it presents a comprehensive analysis of the potential functional and molecular interactions between kynurenic acid and HCAR3 in the context of cancer pathophysiology, which may have significant implications for tumor immunomodulation and the development of new therapeutic strategies.

## 1. Introduction

The hydroxycarboxylic acid receptor (HCAR) family constitutes a subgroup of G-protein-coupled receptors (GPCRs), whose activation has been implicated in a broad spectrum of physiological and pathological processes. The HCARs gene cluster, located on chromosome 12, encodes three paralogous receptors: HCAR1 (GPR81), HCAR2 (GPR109A), and HCAR3 (GPR109B). Notably, HCAR3 expression is unique to humans and select primates closely related to humans, thereby limiting the feasibility of in vivo studies in conventional animal models. Conversely, this phylogenetic specificity presents a unique opportunity to investigate its potential role in human-specific pathologies [1]. HCAR3 is activated by several endogenous and synthetic compounds, including kynurenic acid (KYNA), 3-hydroxyoctanoic acid (3-OH), niacin, 4-(n-propyl)amino-3-nitrobenzoic acid, 3-[bis(thiophen-3-ylmethyl)amino]-1H-pyrazole-5-carboxylic acid, D-phenylalanine, D-tryptophan, and D-kynurenine [2,3,4,5,6,7,8,9].

HCAR3, a multi-pass membrane protein, predominantly localizes to intercellular junctions (The Human Protein Atlas: https://www.proteinatlas.org/ENSG00000255398-HCAR3/subcellular, available on 24 April 2025), with its highest expression in normal human tissues observed in the kidney, liver, and lung [10]. HCAR3 expression has also been confirmed in epidermal keratinocytes, where it modulates key cellular processes, such as proliferation, migration, and mitochondrial respiration. Gene silencing of *HCAR3* alters multiple gene networks essential for preserving cellular identity and promoting proliferation, while concurrently suppressing differentiation. Furthermore, *HCAR3* knockdown impairs keratinocyte motility and bioenergetic function, underscoring its regulatory role in cellular metabolism [11].

HCAR3 engages in signaling pathways that regulate both metabolic and immune functions [1]. Activation by 3-hydroxyoctanoic acid (3-OH) suppresses the release of free fatty acids, thereby indirectly attenuating lipolysis and β-oxidation of fatty acids [12]. HCAR3 modulates intracellular second messengers, particularly cAMP and Ca^2+^, resulting in decreased cAMP concentrations and inhibition of lipolysis. Conversely, activation of Gi-coupled signaling cascades in immune cells by HCAR3 leads to elevated intracellular calcium levels [1,12]. HCAR3 is predominantly expressed in neutrophils, macrophages, monocytes, and basophils, supporting its pleiotropic role in systemic immune regulation. Its activation has been linked to immunomodulatory effects, including attenuation of immune responsiveness and promotion of anti-inflammatory pathways [13].

These findings indicate that HCAR3 may participate in broader immunomodulatory networks, with potential relevance to cancer development and tumor immunosurveillance. Emerging evidence implicates HCAR3 in key oncogenic processes, including cell proliferation, motility, and metabolic reprogramming of tumor cells. Concurrently, the kynurenine pathway, the main pathway of tryptophan metabolism, is frequently dysregulated in multiple cancer types, resulting in altered production of biologically active metabolites. KYNA, an endogenous ligand of HCAR3, represents a significant metabolite of the kynurenine pathway with well-documented immunomodulatory properties. Notably, KYNA has also been shown to modulate cancer cell metabolism, proliferation, survival, and migration, through both direct and indirect mechanisms [14,15,16,17,18].

Accordingly, this review aims to elucidate the potential role of HCAR3 in cancer initiation, progression, and metastasis, and to evaluate whether current evidence confirms its involvement in oncogenesis. Moreover, the review also provides a comprehensive analysis of the potential crosstalk between HCAR3 and the kynurenine metabolic axis within the context of cancer pathophysiology. Particular attention is given to the potential functional interplay between KYNA and HCAR3, including receptor-mediated signaling pathways and their downstream biological effects in tumor cells. Finally, we identify critical gaps in knowledge and propose directions for future investigation.

## 2. HCAR3 in Cancer

Previous studies suggest that HCAR3 may play an important role in the cancer pathophysiology. Differential expression analyses across diverse malignancies have revealed marked alterations in *HCAR3* transcript levels relative to corresponding normal tissues (Table 1). According to RNA-sequencing data, lung squamous cell carcinoma, head and neck squamous cell carcinoma, bladder urothelial carcinoma, cervical squamous cell carcinoma, endocervical adenocarcinoma, and kidney chromophobe were classified into the *HCAR3* expression-enriched group (https://www.proteinatlas.org/ENSG00000255398-HCAR3/cancer, accessed on 24 April 2025). The observed heterogeneity in *HCAR3* expression across tumor types suggests that its functional activity and downstream biological effects may be context-dependent and vary among distinct cancer subtypes.

Further studies indicate HCAR3’s role in regulating cellular processes relevant to cancer biology. The receptor modulates epithelial cell proliferation, migration, and cellular metabolism. Gene silencing of *HCAR3* disrupts transcriptional programs involved in maintaining cellular identity and promoting proliferation and concurrently impairs cell migration and cellular respiration. These observations suggest that HCAR3 may also modulate key biological processes in cancer cells, influencing their growth, invasiveness, and metabolism [10,11]. Notably, *HCAR3* expression has been correlated with clinical outcomes, such as overall survival, in patients diagnosed with colorectal cancer and cervical cancer [19,20]. Accordingly, the following sections will explore the potential roles of HCAR3 in specific cancer entities.

### 2.1. Colorectal Cancer

Colorectal cancer ranks among the most prevalent malignancies and represents a major contributor to cancer-related mortality, largely attributable to its asymptomatic presentation in early stages and its multifactorial etiology involving genetic, environmental, and lifestyle factors [21]. Integrated bioinformatic and experimental analyses have identified *HCAR3* as a potential biomarker implicated in the pathogenesis of colorectal cancer. Yang et al. reported elevated *HCAR3* mRNA levels in colorectal tumor tissue relative to adjacent normal tissue [19]. Moreover, *HCAR3* expression has been confirmed in LoVo colorectal adenocarcinoma cells [22]. *HCAR3* has been identified as one of ten key genes associated with colorectal cancer pathophysiology, alongside *CXCL1*, *CXCL6*, *CXCL8*, *CXCL2*, *CXCL5*, *PPY*, *SST*, *INSL5*, and *NPY1R*. This observation suggests that HCAR3 may play a significant role in cancerogenesis, including colorectal cancer [19]. Interestingly, despite transcriptional upregulation, no significant differences in HCAR3 protein levels were observed between colorectal cancer and normal tissues. Nevertheless, higher *HCAR3* expression correlated positively with improved overall survival [19]. These results may suggest that *HCAR3* expression may be subject to post-transcriptional or post-translational regulatory mechanisms, resulting in maintaining a stable level of protein despite gene overexpression.

**Figure 1:**
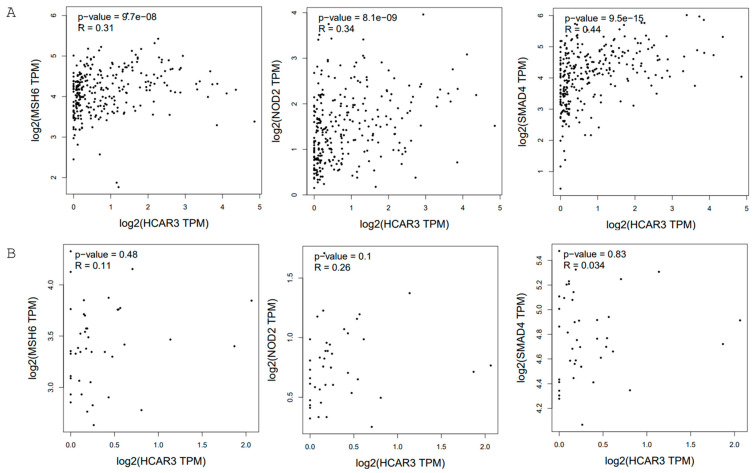
The correlation analysis between *HCAR3* and *MSH6*, *NOD2*, and *SMAD4* in colon adenocarcinoma (**A**) and non-carcinoma control samples (colon adenocarcinoma–normal) (**B**) generated by GEPIA2 (TCGA database; Spearman analysis was calculated using non-log scale; *p* ≤ 0.05 considered significant). Data are shown as TPM values presented at the log-scale axis [25].

Some genetic mutations have been identified as significant contributors to colorectal cancer susceptibility. The predisposing genes include *MSH2*, *MLH1*, *MSH3*, *MSH6*, *NOD2*, *EPCAM*, *PMS2*, *STK11*, *SMAD4*, *BMPR1A*, and others [23,24]. Importantly, GEPIA2-based transcriptomic analysis revealed the correlations between the expression of *HCAR3* and *MSH6*, *NOD2*, and *SMAD4* in colon adenocarcinoma, but not in control tissue (Figure 1). Conversely, the stable level of HCAR3 protein in colorectal tissue may suggest that the biological activity of only one receptor, HCAR3, might not be critical for colorectal cancer progression, but genetic and metabolic signal networks with other genes that increase the risk of developing cancer might play a crucial role. Moreover, Yang et al. suggested that HCAR3 might be considered a prognostic biomarker for colorectal cancer [19].

### 2.2. Breast Cancer

Breast cancer represents the second leading cause of cancer-related mortality among women worldwide, which generates high social and economic costs [26]. Consequently, elucidating novel molecular and metabolic mechanisms contributing to breast cancer pathogenesis remains a key research priority, particularly in the pursuit of innovative therapeutic targets. Notably, McGuire Sams et al. classified *HCAR3* as a putative oncogene in breast cancer [27]. Previous studies demonstrated that HCAR3 is overexpressed in human breast cancer tissues, primary tumor cells, and representative breast cancer cell lines—BT-474, HCC1954, and HCC38—encompassing the major molecular subtypes: progesterone receptor (PR)^+^/estrogen receptor (ER)^+^/HER2^+^, PR^−^/ER^−^/HER2^+^, and triple-negative, respectively [10,27]. *HCAR3* expression in primary breast cancer cells was approximately 50-fold higher in comparison to the MCF12A epithelial breast cell line [10].

Previous studies revealed that HCAR3 in breast cancer is involved in lipid metabolism. Notably, Stäubert et al. proposed that HCAR3 orchestrates lipid and fatty acid metabolic pathways critical for the viability and survival of malignant cells [10]. Interestingly, specific polymorphic variants within the *HCAR3* gene (*HCAR3* c.560G > A (p.R187Q) and *HCAR3* c.1117delC (p.Q373Kfs*82)) might enhance the regulatory activity of the receptor in fatty acid metabolism [27,28,29]. Notably, these specific variants have been associated with increased breast cancer risk [27].

**Figure 2:**
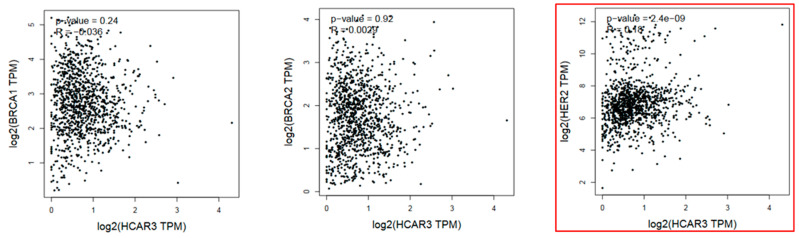
The correlation analysis between *HCAR3* and *BRCA1*, *BRCA2*, and *HER2* in invasive breast carcinoma generated by GEPIA2 (TCGA database; Spearman analysis was calculated using non-log scale; *p* ≤ 0.05 considered significant). Data are shown as TPM values presented at the log-scale axis. Highlighting indicates statistically significant data [25].

Importantly, siRNA-mediated downregulation of *HCAR3* led to reduced viability and the relative ATP levels in BT-474 and HCC1954 breast cancer cells, whereas *HCAR3* overexpression increased viability in these cell lines. Moreover, *HCAR3* silencing triggered cell death in BT-474, HCC1954, and HCC38 breast cancer cells [10]. However, it should be emphasized that the efficacy of siRNA-mediated *HCAR3* silencing varied depending on the incubation duration, serum concentration in culture media, and cellular context [10]. Thus, it cannot be excluded that observed biological effects were also co-regulated by additional factors. In contrast to the gene expression correlations observed in colon adenocarcinoma (Figure 1), no statistically significant associations were detected between *HCAR3* and *BRCA1* or *BRCA2* expression in invasive breast carcinoma. A weak correlation was identified only between *HCAR3* and *HER2* (Figure 2). These results suggest that the possible role of HCAR3 in pathogenesis and progression of breast cancer is not related to the biological activity of the most common breast-cancer-related genes.

### 2.3. Acute Myeloid Leukemia (AML)

Notably, HCAR3 abnormalities were observed in patients with acute myeloid leukemia. The rural Appalachian population exhibits increased genomic instability, resulting in a higher incidence of mutations, including those affecting *HCAR3*. Moreover, the estimated five-year overall survival of this population was decreased in comparison to a control group. It remains unclear whether mutations in *HCAR3* are causally implicated in this outcome, as concurrent mutations in other genes were also identified within this patient cohort. Nonetheless, the observed mutational profile in this population may hold future diagnostic and prognostic relevance [30].

### 2.4. Skin Cancer 

In physiologically normal human skin, HCAR3 is predominantly expressed in epidermal keratinocytes, with its protein detected from the basal through to the granular layers of the epidermis. Within the basal layer, HCAR3 exhibits a dispersed intracellular localization, while in more differentiated layers, it predominantly localizes to the plasma membrane. Notably, *HCAR3* expression is below limits of detection in dermal fibroblasts. In both primary and immortalized keratinocytes, HCAR3 retains functional activity, localizing to the plasma membrane and mediating signaling through Gi proteins [3].

The expression profile of *HCAR3* changes significantly in the context of cancer, particularly in skin squamous cell carcinoma (SCC). While early-stage SCCs do not show a marked increase in *HCAR3* mRNA expression, advanced and invasive lesions demonstrate up to a sixteen-fold increase relative to normal skin tissue. However, in SCC-derived cell lines, HCAR3 exhibits diffuse intracellular localization, and the receptor is nearly non-functional in terms of signaling, in contrast to its activity in normal keratinocytes [3]. The epidermoid carcinoma cell line A-431 is characterized by high HCAR3 expression [3,31]. These findings suggest that HCAR3 may play an important role in skin biology and cancer progression. Its upregulation in advanced SCC, coupled with altered localization and impaired function in cancer cells, suggests a possible contribution to oncogenic signaling pathways perturbed during malignant transformation.

Although the mutations and oncogenic potential of HCAR3 in some types of cancer might be disturbing, individuals with melanoma exhibit markedly lower levels of *HCAR3* gene expression relative to healthy skin samples, according to GEPIA2 database results. Interestingly, *HCAR3* expression was lower in metastatic melanoma than in primary melanoma samples. Accordingly, Scatozza et al. proposed that *HCAR3* expression patterns may serve as a potential biomarker for melanoma progression [32]. Importantly, nicotinamide, a known HCAR3 ligand, has been shown to inhibit proliferation and increase apoptosis in melanoma cells. Moreover, in vivo experiments confirmed the anticancer activity of nicotinamide. Further investigations are warranted to comprehensively elucidate the functional significance of HCAR3 in both cutaneous homeostasis and carcinogenesis.

### 2.5. Other Types of Cancer

HCAR3 dysregulation has also been documented in various other malignancies. Previous studies reported the potential role of HCAR3 in the regulation of immune processes and metabolism. *HCAR3* has been identified as one of the genes used to construct an immune-related gene prognostic index (IRGPI) for esophageal squamous cell carcinoma (ESCC). High IRGPI levels, based on the expression of *CLDN1*, *HCAR3*, *FNBP1L*, and *BRCA2* genes, were associated with unfavorable prognosis in ESCC patients. These findings underscore the potential utility of HCAR3 as a prognostic biomarker in ESCC [33].

Moreover, Li et al. revealed that *HCAR3*, alongside *CXCL9*, *CXCL10*, *CXCL11*, *PPY*, and *LPAR3* genes, was correlated with clinical outcomes in pancreatic cancer [34]. Additionally, survival analysis revealed that *HCAR3* was negatively associated with the survival rate of patients diagnosed with cervical squamous cell carcinoma and endocervical adenocarcinoma [20].

## 3. The Kynurenine Pathway in Cancer

**Figure 3:**
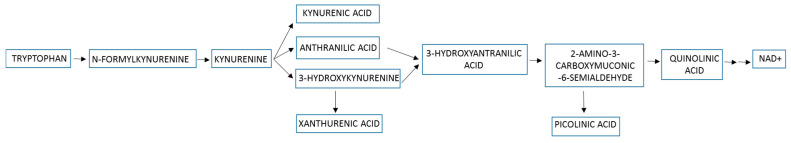
Schematic overview of the kynurenine pathway and its principal metabolic products.

The kynurenine pathway represents the main route of tryptophan metabolism, leading to the formation of the important redox cofactor nicotinamide adenine dinucleotide (NAD^+^) and various bioactive metabolites, including kynurenine, KYNA, anthranilic acid, xanthurenic acid, quinolinic acid, and others (Figure 3) [14,35]. Under physiological conditions, this pathway is stringently regulated and contributes to the maintenance of cellular homeostasis and immune response regulation. However, in the context of cancer, the kynurenine pathway is dysregulated, which may contribute to cancer progression and immunosuppression in the tumor microenvironment. Previous studies have demonstrated that tryptophan depletion, accompanied by elevated levels of immunosuppressive metabolites, such as kynurenine, facilitates immune evasion by tumor cells [36,37,38]. Moreover, upregulation of the rate-limiting enzymes indoleamine 2,3-dioxygenase 1 (IDO1) and tryptophan 2,3-dioxygenase (TDO) has been frequently observed in various malignancies. Furthermore, kynurenine and increased IDO activity have been strongly implicated in carcinogenesis, metastasis, and chemoresistance of cancer cells [14,39,40]. However, recent publications suggest that the relative balance among kynurenine pathway metabolites, rather than levels of individual compounds or enzymatic activities, exerts a more pronounced influence on cellular regulatory mechanisms, including metabolic reprogramming in cancer [41]. Given the growing recognition of metabolic reprogramming as a hallmark of cancer, this review explores a potential interaction network between HCAR3 and its putative endogenous ligand, KYNA.

## 4. The Possible Interactions Between Kynurenic Acid and HCAR3 in Cancer

KYNA, an end-metabolite of the tryptophan degradation cascade, has historically been characterized as a neuroprotective and anticonvulsant compound. However, its presence has also been documented across a range of peripheral tissues and biological fluids (reviewed in [14]). KYNA has been implicated in diverse physiological and pathological processes, including anti-inflammatory modulation, analgesic effects, gastroprotection, atheroprevention, antioxidant activity, and hepatoprotective functions [42,43,44,45,46,47,48]. KYNA has also been detected in tumor tissue, blood, and urine from patients with cancer (reviewed in [14]). Nonetheless, the precise role of KYNA in tumor progression and metastatic dissemination remains incompletely defined. Interestingly, cancer cells may endogenously produce KYNA. Previous studies confirmed the synthesis of KYNA in human gliomas, myeloma, and colon adenocarcinoma cell lines [13,17,49,50]. Available evidence supports the hypothesis that cancer-cell-derived KYNA may serve multiple functional roles within the tumor microenvironment. KYNA may act as an immune response modulator in the tumor microenvironment, contributing to immunosuppression and immune evasion by cancer cells. Additionally, KYNA may directly influence cancer cell biology via activation of specific receptors, including potentially HCAR3, leading to activation of signaling pathways involved in cell survival and proliferation. Moreover, KYNA synthesis may represent an adaptive mechanism of cancer cells, associated with tryptophan metabolism disruptions and cellular homeostasis regulation. However, it is also plausible that enhanced KYNA production merely reflects the global metabolic acceleration characteristic of neoplastic cells, rather than serving a distinct functional role. Comprehensive validation through large-scale, multicenter metabolomic investigations will be required to substantiate this hypothesis.

KYNA may act as a ligand for the HCAR3 receptor, potentially modulating cancer cell functions via the activation of distinct intracellular signaling pathways. Kapolka et al. demonstrated that KYNA activates HCAR3 and may serve as an orthosteric agonist of this receptor [9]. Notably, KYNA exhibits approximately 20-fold greater potency (lower EC_50_) at HCAR3 compared to GPR35, another GPCR targeted by this tryptophan-derived metabolite [9]. However, to date, no robust studies have elucidated the functional consequences of this interaction in cancer cells, and direct experimental evidence supporting this hypothesis remains lacking. Despite decades of investigation into the molecular activity of tryptophan metabolites, the precise mechanisms underlying their biological effects on cancer cells have yet to be clearly defined.

**Figure 4:**
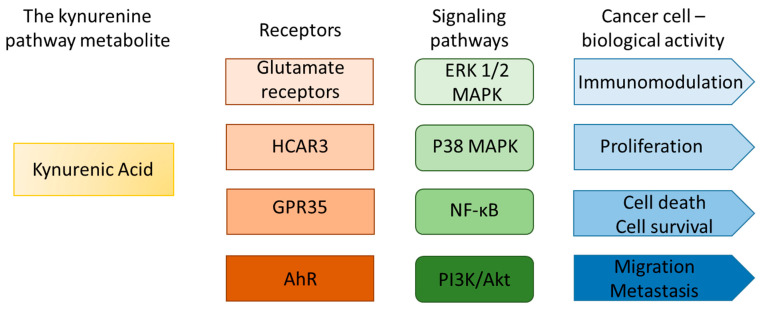
The functional network of the kynurenine pathway metabolite KYNA and cancer cells [9,11,14,15,16,17,18,57,58,60].

Considering the available data and theoretical premises, a functional connection between the kynurenine pathway and HCAR3 receptor in the context of cancer pathogenesis may be hypothesized (Figure 4). First, the molecular structure of KYNA shows similarities to known HCAR ligands, supporting its potential capacity to interact with these receptors [9,51]. Second, both HCAR3 and KYNA are involved in the regulation of inflammatory responses and immune modulation, suggesting a convergence of biological activity through shared signaling pathways [1,52]. Moreover, dysregulation of the kynurenine pathway and altered *HCAR3* expression have been reported across various malignancies, reinforcing the plausibility of their functional interplay in cancer pathophysiology [51,53]. Analysis of TCGA tumor data via the GEPIA2 platform revealed correlations between the expression profile of the *HCAR3* gene and genes coding kynurenine aminotransferases, enzymes directly involved in KYNA synthesis (*CCBL1*, *AADAT*, *CCBL2*, and *GOT2*), particularly in tumors stratified by differential *HCAR3* expression. However, no consistent gene expression pattern emerged from these correlations, suggesting that the potential KYNA–HCAR3 interaction may not be regulated at the transcriptional level or may depend on other regulatory factors. These findings suggest that KYNA–HCAR3 interactions may activate distinct signaling and metabolic pathways, ultimately influencing cancer cell phenotypes and modulating their crosstalk with the tumor microenvironment.

Available literature data suggest that KYNA may directly influence cancer cell biology through activation of specific receptors and associated signaling pathways. Proposed mechanisms include the modulation of pathways involved in cell survival, proliferation, migration, and metabolic regulation. Notably, KYNA has been reported to interact with a diverse array of receptor groups, including HCAR3, glutamate receptors (N-methyl-d-aspartate receptor (NMDA), α-amino-3-hydroxy-5-methyl-4-isoxazolepropionic acid receptor (AMPA), and kainate receptor), α-7 nicotinic acetylcholine receptor (α7 nAChR), aryl hydrocarbon receptor (AhR), and GPR35 [9,54,55,56,57,58]. Additionally, Kapolka et al. proposed that KYNA may also interact with adrenoceptor alpha-2B (ADRA2B) [9]. These findings imply that the biological activity of KYNA in cancer cells likely results from a complex network of interactions involving multiple receptor types and signaling pathways.

Potential molecular mechanisms by which the KYNA–HCAR3 axis may influence cancer biology include the activation of key intracellular signaling pathways, particularly the extracellular signal-regulated kinase 1/2 (ERK1/2) pathway and the NF-kB pathway. KYNA has been linked to the modulation of ERK1/2 activity, which plays a central role in regulating cancer cell proliferation, survival, and metastasis. Notably, KYNA has been shown to inhibit the activation of ERK1/2, p38 MAPK, and Akt kinases in colon cancer and renal cancer cells [15,16]. Interestingly, KYNA did not affect ERK1/2 activation in SK-MEL-3 melanoma cells under basal conditions; however, following UVB exposure, a stimulatory effect of KYNA on ERK1/2 was observed [18]. Concurrently, HCAR3 activation has also been associated with ERK1/2 pathway engagement, suggesting that KYNA may exert some of its effects via HCAR3-mediated signaling. Furthermore, activation of the NF-kB pathway via the KYNA–HCAR3 axis may promote the transcription of genes involved in cell survival, angiogenesis, invasiveness, and immune response modulation, collectively contributing to cancer progression [14,38,51,52,59].

Direct interaction of KYNA with signaling pathways may influence the regulation of cancer cell proliferation. In vitro studies have demonstrated the antiproliferative activity of KYNA toward colon cancer, renal cancer, melanoma, and glioblastoma cells [14,15,17,18]. A comparable inhibitory effect was observed in melanoma A375 and SK-MEL-28 cells exposed to nicotinamide, another HCAR3 ligand [32]. Importantly, HCAR3 is also involved in the proliferation, differentiation, and survival of normal cells. The silencing of *HCAR3* in human primary keratinocytes resulted in reduced migration and impaired metabolic activity [11].

It should be underlined that the KYNA–HCAR3 functional network may exert both direct and indirect effects on cancer cells, particularly through the modulation of immune responses. HCAR3 is highly expressed in various immune cell types, including neutrophils, monocytes, and basophils, but also in hematopoietic tissues, such as the bone marrow, and the spleen [13]. Notably, previous studies revealed that activation of HCAR3 may decrease the response of the immune system and promote anti-inflammatory effects [61,62]. Moreover, HCAR3 participates in the regulation of innate and adaptive immune pathways by cAMP-dependent signaling mechanisms [63]. Recent studies identified a functional interplay between HCAR3 and AhR in cancer [60]. The AhR- and HCAR3-dependent pathways have been implicated in the microbiota-mediated immunomodulation of the tumor microenvironment in gastric cancer [60]. Both HCAR3 and AhR may serve as biosensors for tryptophan metabolites. Moreover, KYNA may play a crucial role in the regulation of the local immune response to cancer. Its documented antioxidant activity may further contribute to anti-inflammatory effects [64]. Conversely, the opposite properties of KYNA and another tryptophan metabolite, kynurenine, are crucial to maintain homeostasis and a state of immune balance. Therefore, KYNA, as a ligand of AhR and GPR35, may indirectly affect immune balance and be involved in tumor immune escape [51,53].

## 5. HCAR3 and Kynurenic Acid in Cancer: Fact or Hypothetical Interaction?

Due to the limited number of published studies, it is currently not possible to definitively determine whether the interaction between HCAR3 and KYNA plays a role in cancer initiation, progression, or metastasis. It is established that KYNA acts as a ligand for HCAR3, activating this receptor at concentrations lower than those required for GPR35—the previously identified KYNA-responsive GPCR [9]. Notably, HCAR3 is considered a metabolite sensor, and metabolic dysregulation, including tryptophan metabolism, is a hallmark of cancer pathophysiology [1,41]. Analysis of the available literature and bioinformatic data reveals numerous overlapping features in the molecular mechanisms and cellular effects associated with HCAR3 and KYNA in cancer. However, these observations require further validation through advanced molecular, in vitro, and clinical investigations to clarify the HCAR3–KYNA interaction network and its potential contribution to carcinogenesis.

The missing pieces of the puzzle in the HCAR3–KYNA functional network and directions for future research include the following:

- Most studies to date have focused on *HCAR3* gene expression in cancer, rather than its functional activity. Notably, overexpression of *HCAR3* is not always correlated with the protein HCAR3 level; however, the regulatory mechanism of this phenomenon has not been studied. Further studies are necessary to indicate post-transcriptional or post-translational regulations involved in this process.
- To validate the hypothesis concerning the role of HCAR3 and KYNA in carcinogenesis, large-scale, multicenter studies employing standardized research protocols and statistical approaches are essential. Available data do not allow us to determine whether the modified expression level of *HCAR3* in cancer enhances cancer progression or whether it is a nonspecific effect of altered metabolism of tumor cells. Is there functional compensation at the protein level between HCAR3 and other HCAR receptors?
- Previous research focused only on the interaction between HCAR3 and KYNA without considering the broader context of the kynurenine pathway and other biologically active tryptophan metabolites. It should be verified whether the suggested functional interaction concerns only HCAR3 and KYNA, or whether we should consider a broader metabolic network. Is it possible to verify the molecular and biological effects of KYNA-mediated HCAR3 activation in cancer cells, taking into account the number of KYNA-activated receptors, signaling pathways, and cell cycle regulators? Unfortunately, the lack of HCAR3 antagonists, gene silencing efficiency, occurrence of paralogs, and expression of HCAR3 only in mammalian cells are significant obstacles to carrying out further advanced research.
- The role of HCAR3 should be considered not only as a direct effect on cancer cells, but also in more complex interactions with the tumor microenvironment and the immune system.

## 6. Conclusions

The body of evidence and research hypotheses presented in this article suggest a potential link between the HCAR3 receptor and KYNA in the context of cancer pathophysiology. The hypothesis that KYNA may serve as a functional ligand for HCAR3, affecting cancer cell biology and modulating the immune response in the tumor microenvironment, represents a novel, interesting research direction. This concept may enhance our understanding of molecular and metabolic mechanisms of cancer progression and identify specific molecular targets for innovative therapeutic strategies.

Elucidating the potential HCAR3–KYNA axis in cancer may have significant clinical and therapeutic implications, as HCAR3 may represent a new prognostic and predictive biomarker in various cancer types, potentially facilitating personalized treatment strategies. Additionally, modulation of HCAR3 activity and/or the kynurenine pathway may represent a new therapeutic strategy in cancer treatment. However, further molecular, in vitro, and clinical investigations are warranted to substantiate these preliminary findings. The KYNA–HCAR3 interactions should be considered as one component within a broader network of signaling events involving receptors, intracellular pathways, cell cycle regulators, and metabolic processes in cancer cells. At the current stage of evidence, most of the proposed interactions between HCAR3 and the kynurenine pathway remain speculative and require experimental validation before being regarded as established mechanisms.

## Figures and Tables

**Table 1:** RNA expression of the *HCAR3* gene in cancers retrieved from The Human Protein Atlas. *HCAR3* expression shows RNA-seq data from The Cancer Genome Atlas (TCGA) and the International Cancer Genome Consortium (validation cohorts). Data are shown as median pTPM values (https://www.proteinatlas.org/ENSG00000255398-HCAR3/cancer, available on 24 April 2025).

	Type of Cancer	pTPM Cancer	pTPM Validation
Central nervous system cancer	Glioblastoma multiforme	0.2 (N = 141)	0.4 (N = 58)
Lung cancer	Lung adenocarcinoma	1.5 (N = 497)	2.8 (N = 105)
	Lung squamous cell carcinoma	6.5 (N = 489)	11.3 (N = 68)
Gastrointestinal cancer	Colon adenocarcinoma	0.5 (N = 254)	3.6 (N = 486)
	Rectum adenocarcinoma	0.4 (N = 88)	2.6 (N = 207)
	Stomach adenocarcinoma	0.5 (N = 346)	No data available
	Liver hepatocellular carcinoma	0.2 (N = 362)	0.5 (N = 231)
	Pancreatic adenocarcinoma	0.5 (N = 176)	1.5 (N = 80)
Urinary tract cancers	Bladder urothelial carcinoma	8.1 (N = 169)	No data available
	Kidney chromophobe	1.2 (N = 64)	No data available
	Kidney renal clear cell carcinoma	0.2 (N = 521)	0.4 (N = 100)
	Kidney renal papillary cell carcinoma	0.1 (N = 282)	No data available
Cancers of the male reproductive system	Prostate adenocarcinoma	0.4 (N = 480)	No data available
	Testicular germ cell tumor	0.9 (N = 133)	No data available
Cancers of the female reproductive system	Cervical squamous cell carcinoma and endocervical adenocarcinoma	7.3 (N = 283)	No data available
	Ovary serous cystadenocarcinoma	0.2 (N = 349)	0.2 (N = 81)
	Uterine corpus endometrial carcinoma	0.1 (N = 176)	No data available
	Skin cutaneous melanoma	0.1 (N = 99)	No data available
	Breast invasive carcinoma	0.7 (N = 1022)	No data available
	Head and neck squamous cell carcinoma	10.5 (N = 492)	No data available

## Abbreviations

α7 nAChR	α-7 nicotinic acetylcholine receptor
AhR	aryl hydrocarbon receptor
AML	acute myeloid leukemia
AMPA	α-amino-3-hydroxy-5-methyl-4-isoxazolepropionic acid
ESCC	esophageal squamous cell carcinoma
ER	estrogen receptor
ERK	extracellular signal-regulated kinase
HCAR	hydroxycarboxylic acid receptor
GPCR	G-protein-coupled receptor
IDO	indoleamine 2,3-dioxygenase
KYNA	kynurenic acid
NAD	nicotinamide adenine dinucleotide
NMDA	N-methyl-d-aspartate receptor
3-OH	3-hydroxyoctanoic acid
PR	progesterone receptor
TCGA	The Cancer Genome Atlas
TDO	tryptophan 2,3-dioxygenase
